# Linear growth faltering and the role of weight attainment: Prospective analysis of young children recovering from severe wasting in Niger

**DOI:** 10.1111/mcn.12817

**Published:** 2019-04-29

**Authors:** Sheila Isanaka, Matt D.T. Hitchings, Fatou Berthé, André Briend, Rebecca F. Grais

**Affiliations:** ^1^ Departments of Nutrition and Global Health and Population Harvard T.H. Chan School of Public Health Boston United States; ^2^ Department of Epidemiology Harvard T.H. Chan School of Public Health Boston United States; ^3^ Department of Research Epicentre Paris France; ^4^ Epicentre Niger; ^5^ Center for Child Health Research University of Tampere School of Medicine Tampere Finland; ^6^ Department of Nutrition, Exercise and Sports, Faculty of Science University of Copenhagen Copenhagen Denmark

**Keywords:** child malnutrition, linear growth, Niger, stunting, wasting, weight gain

## Abstract

Efforts to reduce the impact of stunting have been largely independent of interventions to reduce the impact of wasting, despite the observation that the conditions can coexist in the same child and increase risk of death. To optimize the management of malnourished children—who can be wasted, stunted, or both—the relationship between stunting and wasting should be elaborated. We aimed to describe the relationship between concurrent weight and height gain during and after rehabilitation from severe wasting. We conducted a secondary analysis of a randomized trial for the outpatient treatment of severe wasting, including 1,542 children who recovered and were followed for 12 weeks. We described the overlap of stunting and severe wasting and the change in stunting over time. We showed the relationship between concurrent weight and height gain using adjusted generalized estimating equations and calculated the mean rate of change in weight‐for‐height *z* score (WHZ) and height‐for‐age *z* score (HAZ) during and after rehabilitation. At baseline, 79% (*n* = 1,223/1,542) and 49% (*n* = 757/1,542) of children were stunted and severely stunted, respectively. Prevalence increased over time among children <24 months. During rehabilitation when weight was not yet fully recovered, we found rapid WHZ gain but limited HAZ gain. Following successful rehabilitation, WHZ gain slowed. The rate of HAZ gain was negative after rehabilitation but increased relative to the period during treatment. The potential relationship between weight and height gain calls for increased coverage of wasting treatment to not only prevent child mortality but also reduce linear growth faltering.

**Key messages**

- Efforts to reduce the impact of stunting have been largely independent of interventions to reduce the impact of wasting, despite the observation that both can conditions coexist in the same child and increase the risk of death.
- In this population of severely wasted children, we found a high burden of stunting and severe stunting at admission. Recovery in linear growth was overall limited, and height attainment was modulated by adequate weight‐for‐height. During rehabilitation when weight was not yet fully recovered, we found rapid weight‐for‐height *z* score (WHZ) gain but limited height‐for‐age *z* score (HAZ) gain. Following successful rehabilitation, WHZ gain slowed whereas HAZ gain increased relative to the period before recovery.
- The potential relationship between weight and height gain calls for increased coverage of wasting treatment to not only prevent child mortality but also reduce linear growth faltering.

**Abbreviations**
CI: confidence interval
HAZ: height‐for‐age *z* score
MUAC: mid‐upper arm circumference
OR: odds ratio
RUTF: ready‐to‐use therapeutic food
SAM: severe acute malnutrition
WHZ: weight‐for‐height *z* score

## 1 INTRODUCTION

Linear growth faltering in children, characterized by falling below the height‐for‐age trajectory of the World Health Organization (WHO) Child Growth Standards, has been the focus of increasing policy, program, and research attention (de Onis & Branca, 2016; Galasso & Wagstaff, 2016; World Bank, 2014). In 2014, there were 162 million children worldwide who were stunted (defined as being at least two standard deviations [SDs] below the median height‐for‐age), and the WHO Global Targets ambitiously aim to reduce this number by 40% by 2025 (WHO, 2014). Children who are stunted have been shown to be at increased risk of infectious disease, impaired cognitive and physical development, and death (Black et al., 2008; Dewey & Begum, 2011; Grantham‐McGregor et al., 2007; Prendergast & Humphrey, 2014).

Linear growth faltering and stunting are considered a manifestation of chronic undernutrition caused by a number of complex factors, including chronic nutrient deficiency and exposure to poor environmental conditions. Programs to prevent stunting in the first 1,000 days of life have included interventions to improve maternal nutrition; infant and young child feeding practices; care‐seeking behaviour; and water, sanitation, and hygiene (Bhutta et al., 2008; Black et al., 2013; Luby et al., 2018; Null et al., 2018). However, likely due to the multitude of and possible interaction between, risk factors for stunting, evidence on the effectiveness of individual interventions has been mixed (Dewey, 2016; Goudet, Griffiths, Bogin, & Madise, 2017; Subramanian, Mejia‐Guevara, & Krishna, 2016).

To date, action to reduce the impact of stunting has been independent of interventions targeting wasting, an acute form of undernutrition identified when a child falls below the WHO's weight‐for‐height standard. Wasting is predominantly caused by acute nutrient deficiency and infection but shares many causal pathways with stunting (Briend, Khara, & Dolan, 2015; Martorell & Young, 2012). Wasting and stunting can often coexist in the same child (Khara, Mwangome, Ngari, & Dolan, 2017). Although each condition is associated with an increased risk of death, it has been shown that the risk is multiplicative when such anthropometric deficits are concurrent (McDonald et al., 2013).

To optimize the management of malnourished children—who can be wasted, stunted, or both—the relationship between stunting and wasting should be elaborated. The association between weight and linear growth in children has been looked at previously, but results have varied by study design and setting (Doherty et al., 2001; Khara & Dolan, 2014; Richard, Black, & Checkley, 2012; Walker & Golden, 1988). We used prospective data from children being treated for severe wasting in Niger to better understand the relationship between concurrent weight and height gain across a wide within‐person range of weight during and after rehabilitation.

## 2 PARTICIPANTS AND METHODS

### 2.1 Study population and procedures

We used prospective data from a randomized trial of routine amoxicillin in the outpatient treatment of severe wasting. Details of the parent study, including study enrolment, inclusion and exclusion criteria, and study procedures have been described in detail elsewhere (Isanaka et al., 2016). Briefly, a total of 2,412 children presenting with uncomplicated severe acute malnutrition (SAM) in Niger were enrolled in outpatient treatment and randomly assigned to receive either amoxicillin or placebo for 7 days (Figure S1). A child was considered to have uncomplicated SAM and be eligible for outpatient treatment if aged between 6 and 59 months; weight‐for‐height *z* score (WHZ) < −3 according to the 2006 WHO Growth Standards and/or mid‐upper arm circumference (MUAC) <115 mm; sufficient appetite according to a test feeding of ready‐to‐use therapeutic food (RUTF); and absence of clinical complications requiring hospitalization, including bipedal oedema. All children received standard outpatient care during treatment, including weekly anthropometric assessment, physical exam, and distribution of RUTF. Children were followed until program discharge (minimum 3 weeks and maximum 8 weeks) as per Médecins Sans Frontières and Government of Niger guidelines and asked to return for additional study visits at 4, 8, and 12 weeks after enrolment for anthropometric assessment and physical exam as per the parent trial protocol. Children who had clinical complications and/or weight loss of more than 5% between consecutive visits, or who had no weight gain for 2 weeks, were transferred to inpatient care and censored from the study. Nutritional recovery was defined as having WHZ > −2 on two consecutive visits and MUAC >115 mm, with no complications or oedema. This secondary analysis was restricted to the 1,542 children who recovered from SAM, as defined above (mean 11.8 weeks with 11,855 total observations). The parent study protocol was approved by the Comité Consultatif National d'Éthique, Niger and the Comité de Protection des Personnes, Île‐de‐France XI, Paris.

### 2.2 Statistical analysis

To describe the overlap of stunting and severe wasting in this population at admission, we calculated the proportion of children who were stunted (defined as having height‐for‐age *z* score [HAZ] < −2), severely wasted (defined by WHZ < −3 and/or MUAC <115 mm), or both. To describe the burden and change in stunting and severe stunting (defined as HAZ < −3) over the 12‐week follow‐up period, we calculated and compared the prevalence of stunting and severe stunting at admission and at end of follow‐up. Significant differences in the prevalence of stunting and severe stunting at admission and the end of follow‐up, overall, and by age (±24 months), were assessed using the chi‐square test.

We next described the response to treatment, characterized by weight gain and change in WHZ and HAZ and time to recovery by stunting status at admission. Mean weight gain (g/kg/day) and time to recovery (days), with associated 95% confidence intervals (CI), by stunting status, were estimated using linear regression. Mean rate of change in WHZ, MUAC, and HAZ and 95% CIs over time were estimated using generalized estimating equations and are presented relative to the end of treatment to differentiate patterns of growth during versus after the period of weight rehabilitation. We finally estimated the association between WHZ at admission and overall change in HAZ over 12 weeks using linear regression. All models were adjusted for age, sex, and parent trial regimen and tested for statistical differences by child age (±24 months), sex, and admission criterion (low WHZ only vs. low MUAC only vs. both).

## 3 RESULTS

### 3.1 Burden and change over time of stunting in the study population

At SAM treatment initiation, nearly four in five children (*n* = 1,223/1,542, 79%) were stunted and 49% severely stunted (*n* = 757/1,542). The lowest burden was among those children identified with WHZ < −3 only (74%), compared with 81% stunted among children identified with MUAC <115 mm only and 80% identified with both MUAC <115 mm and WHZ < −3. Stunted and wasted children, compared with those nonstunted, were on average older (18.6 months vs. 12.7 months) and more likely to be male (50.7% vs. 39.5%) and have lower MUAC at admission (113 mm vs. 115 mm; Table 1).

**Table 1:** Baseline characteristics of study population

Characteristic	Total (*n* = 1,542)	Severely wasted only (*n* = 319)	Severely wasted and stunted (*n* = 1,223)	*p* value^1^
Sociodemographic characteristics				
Child age (month)	17.4 ± 8.6	12.7 ± 6.9	18.6 ± 8.5	<0.0001
Mother age (year)	27.2 ± 6.7	26.8 ± 6.2	27.3 ± 6.8	0.34
Male sex	746 (48.4)	126 (39.5)	620 (50.7)	0.0004
Maternal education (primary school complete or higher)	236 (15.3)	51 (16.0)	185 (15.1)	0.70
No. of household members	7.4 ± 3.9	7.3 ± 3.8	7.4 ± 3.9	0.92
Anthropometric data				
WHZ	−3.0 ± 0.6	−3.0 ± 0.6	−3.0 ± 0.6	0.18
WHZ < −3	878 (56.9)	195 (61.1)	683 (55.9)	0.09
MUAC (mm)	112.8 ± 4.1	113.7 ± 3.5	112.6 ± 4.2	<0.0001
MUAC <115 mm	1,193 (77.4)	228 (71.5)	965 (78.9)	0.005
HAZ	−3.0 ± 1.2	−1.4 ± 0.5	−3.4 ± 1.0	<0.0001
Clinical characteristics and medical history				
Haemoglobin <11.0 g/dl	1,150 (74.6)	215 (67.4)	935 (76.5)	0.001
Rapid diagnostic test positive for malaria	904 (58.6)	178 (55.8)	726 (59.4)	0.25
Axillary temperature >38.5°C	72 (4.7)	17 (5.3)	55 (4.5)	0.53
Signs of infection in previous 24 hr				
Diarrhoea	495 (32.1)	108 (33.9)	387 (31.6)	0.45
Vomiting	91 (5.9)	32 (10.0)	59 (4.8)	0.0004
Cough	273 (17.7)	65 (20.4)	208 (17.0)	0.16
Seen at health facility in previous 30 days	352 (22.8)	80 (25.1)	272 (22.2)	0.28
Child currently breastfeeding	918 (60.0)	258 (80.1)	660 (54.0)	<0.0001

Values are *n* (%) or mean ± *SD*

1 *p* value from a *t* test for continuous variables and from a chi‐square test of independence for categorical variables.

Overall, recovery in linear growth in this population was limited, with mean SD HAZ decreasing over time from −3.0 (1.2) at admission to −3.3 (1.1) at end of follow‐up. The prevalence of stunting and severe stunting by age group at admission and the end of the 12‐week follow‐up period is shown in Table 2. At admission, both stunting and severe stunting were higher among children ≥24 months of age, compared with children <24 months. No increase in stunting or severe stunting was noted among children ≥24 months. In contrast, among younger children, both stunting and severe stunting significantly increased during the 12‐week follow‐up.

**Table 2:** Prevalence of stunting and severe stunting over 12 weeks from admission among children 6–23 months and 24–59 months of age

	Prevalence at baseline	Prevalence at end of follow up
6–23 months of age		
Stunting	76.1%	86.8%*
Severe stunting	43.6%	55.5%*
		
24–59 months of age		
Stunting	94.8%	94.4%
Severe stunting	75.3%	78.3%

* *P* for difference between prevalence at baseline versus end of follow‐up <0.05.

### 3.2 Response to treatment

Response to treatment was independent of stunting status at admission. There was no difference in weight gain among stunted versus nonstunted children during treatment (5.9 g/kg/day vs. 5.7 g/kg/day; difference 0.24, 95% CI: [−0.07, 0.56]) or after treatment (0.9 g/kg/day vs. 0.9 g/kg/day; difference 0.1, 95% CI: [−0.1, 0.3]). Mean time until recovery was also independent of stunting status (30.6 days vs. 30.1 days; difference 0.5, 95% CI: [−0.8, 1.7]).

**Figure 1:**
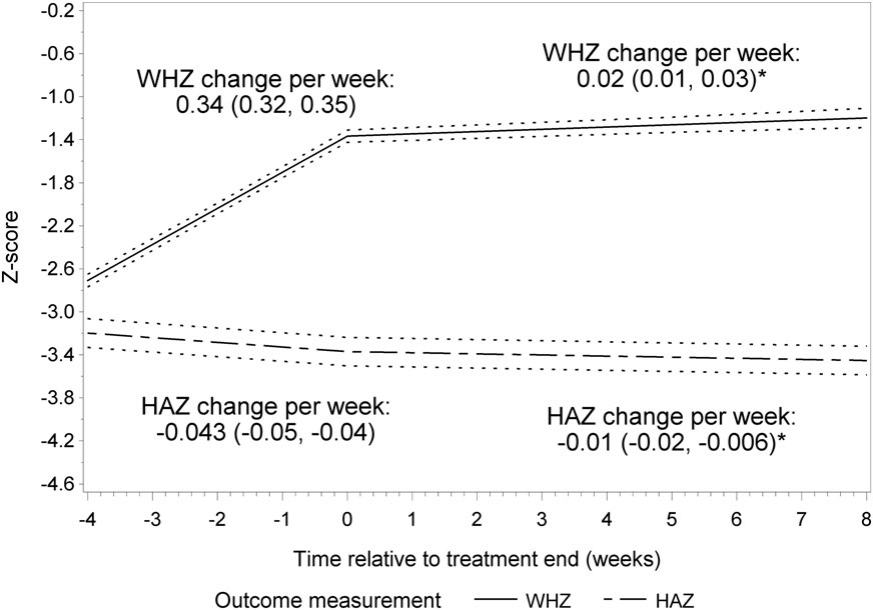
Mean change in weight‐for‐height *z* score (solid) and height‐for‐age *z* score (dashed) during and following treatment estimated from generalized estimated equations, with 95% confidence intervals shown as dotted lines, for an 18‐month‐old boy in the placebo group. A * denotes that post‐treatment change is significantly different from pretreatment change (*p* < .05)

Overall, WHZ at baseline was significantly associated with a 0.08‐unit (95% CI: [−0.05, 0.12]) increase in HAZ over 12 weeks. During rehabilitation (e.g., while weight had not yet fully recovered), we observed rapid WHZ gain but limited HAZ gain (Figure 1 and Table S1). WHZ gain during rehabilitation was greatest among children admitted with low WHZ and low MUAC (Figure S2) and similar to the overall pattern of MUAC gain over time (Figure S3). Following successful rehabilitation and recovery of weight, the rate of WHZ gain slowed. The rate of HAZ gain was negative after rehabilitation but increased relative to the period during treatment. Among children who gained HAZ during follow‐up (*n* = 366), we found recovery in linear growth was more often initiated after discharge (77%), compared with during rehabilitation (23%).

## 4 DISCUSSION

In this population of severely wasted children, we found a high burden of stunting and severe stunting at admission. Stunting at admission, however, did not impair response to treatment, as there was no difference in weight gain or time to recovery by stunting status. We found that recovery in linear growth was overall limited in this population, marked by a mean decrease in HAZ over time, and HAZ gain was modulated by WHZ/nutritional recovery. During treatment although children were undergoing rapid weight gain and recovering WHZ, there was limited gain in HAZ and stunting increased on average. After nutritional rehabilitation, WHZ gain slowed whereas HAZ gain increased relative to the period before recovery.

The burden of stunting and the concurrence of stunting and severe wasting, observed in this population, have not been well described but were particularly high: Among severely wasted children, 79% were stunted, and 49% were severely stunted. In an analysis of nationally representative data from 84 countries, Niger was found to have the highest rate of concurrence of wasting and stunting globally (8%, 95% CI: [7.2, 8.9]) (Khara et al., 2017). The high rate of concurrence observed in our analysis underscores how concurrent severe wasting and stunting can be concentrated in the most vulnerable populations of severely malnourished children. In light of the increased risk of death associated with concurrence (McDonald et al., 2013), children who are both stunted and severely wasted should be prioritized for therapeutic treatment. Notably, our analysis confirms that stunting at admission was not associated with an impaired response to wasting treatment and therefore should not limit the scaling up of wasting treatment among these high‐risk children.

Our results further support the hypothesis that recovery in linear growth depends on adequate weight, which has been previously observed in malnourished populations. Among children recovering from severe wasting, historical data from Walker and Golden (Walker & Golden, 1988) showed that of those children who had linear catch‐up growth, two thirds started to gain height only after reaching a WHZ ≥ 85% of the National Center for Health Statistics median. We similarly found gain in HAZ to begin only after nutritional recovery among 77% children who had experienced recovery in linear growth. More recent studies have failed to show an association between treatment with RUTF and stunting during (Khara & Dolan, 2014) or up to 12 months after discharge (Kerac et al., 2014). Authors have suggested this lack of relationship may be due to a suboptimal RUTF formulation that is limited in some specific micronutrients needed to support bone growth (Khara & Dolan, 2014), but linear catch‐up growth may have been impeded by insufficient weight of the malnourished populations under study. Community‐based studies on the use of other ready‐to‐use foods to reduce seasonal peaks in wasting found modest gains in HAZ with short‐term intervention (Huybregts et al., 2012; Isanaka et al., 2009; Isanaka et al., 2010). This result was not confirmed in all settings (Grellety et al., 2012; Thakwalakwa et al., 2012) but attainment are possible when children start with adequate weight and fat mass.

Studies to describe the relationship between weight and height attainment among children with a wider range of WHZ have also been conducted. Results from cross‐sectional studies are mixed, but those from longitudinal studies are largely consistent with the hypothesis of a direct relationship. Richard et al. (Richard et al., 2012) analysed eight cohort studies (*n* = 1,599) and found infants with recent episodes of wasting had lower HAZ scores, whereas increases in WHZ in the previous 6 months were positively associated with linear growth at 18 and 24 months. These results suggest that periods of wasting are associated with decreased linear growth and that recovery from wasting may allow HAZ to be subsequently recovered. Walker et al. (Walker, Grantham‐McGregor, Himes, & Powell, 1996) also link variations in WHZ with linear growth, finding that gain in WHZ in a 6‐month interval positively predicted linear growth in the following interval. Similarly, children in Nepal with low WHZ at the beginning of the study period had lower height velocity than children with normal WHZ (Costello, 1989), suggesting again that low weight may impede height gain. Finally, in several longitudinal studies exploring the relationship of weight and height by season, the lowest linear growth was found to follow periods of lowest weight gain so that height was only attained when WHZ was regained at the end of the hunger season (Brown, Black, & Becker, 1982; Maleta, Virtanen, Espo, Kulmala, & Ashorn, 2003).

Mechanisms underlying the relationship between weight and height growth are being explored. Fat and bone, which secrete hormones such as leptin, can modulate bone density and height attainment (Gat‐Yablonski et al., 2004; Karsenty, 2006); reduced stores of leptin‐producing fat cells among wasted children may therefore be one mechanism through which insufficient weight could restrict bone and linear growth. Evidence among children supports the hypothesis that linear catch‐up growth may be limited by insufficient leptin concentration (Büyükgebiz, Öztürk, Yilmaz, & Arslan, 2004). Although such evidence supports a direct relationship between weight and height attainment, we caution against the interpretation that wasting would be the primary cause of linear growth faltering. Wasting and linear growth faltering share a number of common causes, which can contribute to their concurrence (Martorell & Young, 2012). Many other factors, however, independently contribute to the development of linear growth faltering. The analysis of eight longitudinal cohorts found that stunting was more common than can be explained by previous wasting alone (Richard et al., 2012), with environmental factors, dietary factors, and infectious disease also likely playing an independent role. A meta‐analysis of 19 birth cohorts suggested a large proportion (20%) of stunting in fact had its origins in the foetal period, showing a strong relationship between being small for gestational age and preterm and stunting later in life (Christian et al., 2013). Stunting is also observed in populations where wasting is uncommon (Chotard, Mason, Oliphant, Mebrahtu, & Hailey, 2010; Waterlow, 1974) and with a high overweight prevalence (Popkin, Richards, & Montiero, 1996), suggesting fat stores may be necessary for linear growth but not sufficient. Other nutrients, such as sulfur, phosphorus, calcium, magnesium, copper, and Vitamins D, K, and C are required in higher amounts for skeletal growth than for growth of other lean tissues and may also be important for linear growth in children (Golden, 2009).

The strength of our study lies in the longitudinal design with a large sample of children and average of 7.7 observations per child. In particular, we were able to observe the same children during treatment and after nutritional rehabilitation, providing a large within‐person range of WHZ for analysis. This provided a unique opportunity to assess the weight‐height relationship during two distinct periods of weight sufficiency. There are however, limitations to the data. First, the parent trial included only children with uncomplicated SAM; the relationship between weight and height gain among less malnourished children and those with clinical complications was therefore not addressed here. Second, the relative contribution of lean and fat tissue during and after recovery from SAM was not assessed but would be important to better understand the dynamics of the relationship between wasting and stunting. Finally, the length of follow‐up was limited to 12 weeks, and it was therefore not possible to examine the long‐term relationship between weight and height gain after nutritional rehabilitation. A recent analysis comparing height attainment in wasted children with sibling and community controls up to 7 years post‐discharge suggested that early weight deficits may have significant effects on height attainment in the long‐term, regardless of the potential for catch‐up growth following rehabilitation (Lelijveld et al., 2016).

In conclusion, we found a high burden of concurrent stunting and severe wasting in this population and provide evidence to support a direct relationship between weight and height attainment, in which inadequate weight in a malnourished child is associated with slowed recovery in linear growth. This slowing of linear growth during a period of severe wasting suggests an under‐appreciated contribution of wasting to stunting. Such a relationship calls for increased coverage and integration of wasting treatment into stunting prevention programs. Increased access to wasting treatment may not only prevent child mortality due to acute malnutrition but also reduce linear growth faltering and mortality associated with multiple anthropometric deficits.

## CONFLICT OF INTEREST

All authors declare that they have no conflict of interests.

## CONTRIBUTIONS

SI and RFG designed the study; FB supported the data collection; MDTH conducted the statistical analysis; SI and MDTH were responsible for the first draft of the manuscript. All authors contributed to the interpretation of data, critically reviewed the manuscript, and decided to publish the paper.

## Supporting information

Table S1. Mean change in weight‐for‐height Z and height‐for‐age Z during and after treatment among boys and girls 6–23 months and 24–59 months of age

Data S1. Supporting information

Data S2. Supporting information

Data S3. Supporting information

## References

[mcn12817-bib-0001] Bhutta, Z. A. , Ahmed, T. , Black, R. E. , Cousens, S. , Dewey, K. , Giugliani, E. , … Shekar, M. (2008). What works? Interventions for maternal and child undernutrition and survival. The Lancet, 371, 417–440. 10.1016/S0140-6736(07)61693-6 18206226

[mcn12817-bib-0002] Black, R. E. , Allen, L. H. , Bhutta, Z. A. , Caulfield, L. E. , de Onis, M. , Ezzati, M. , … Rivera, J. (2008). Maternal and child undernutrition: global and regional exposures and health consequences. The Lancet, 371, 243–260. 10.1016/S0140-6736(07)61690-0 18207566

[mcn12817-bib-0003] Black, R. E. , Victora, C. G. , Walker, S. P. , Bhutta, Z. A. , Christian, P. , de Onis, M. , … Uauy, R. (2013). Maternal and child undernutrition and overweight in low‐income and middle‐income countries. The Lancet, 382, 427–451. 10.1016/S0140-6736(13)60937-X 23746772

[mcn12817-bib-0004] Briend, A. , Khara, T. , & Dolan, C. (2015). Wasting and stunting—Similarities and differences: Policy and programmatic implications. Food and Nutrition Bulletin, 36, S15–S23. 10.1177/15648265150361S103 25902610

[mcn12817-bib-0005] Brown, K. H. , Black, R. E. , & Becker, S. (1982). Seasonal changes in nutritional status and the prevalence of malnutrition in a longitudinal study of young children in rural Bangladesh. The American Journal of Clinical Nutrition, 36, 303–313. 6808822

[mcn12817-bib-0006] Büyükgebiz, B. , Öztürk, Y. , Yilmaz, S. , & Arslan, N. (2004). Serum leptin concentrations in children with mild protein‐energy malnutrition and catch‐up growth. Pediatrics Int, 46, 534–538. 10.1111/j.1442-200x.2004.01951.x 15491379

[mcn12817-bib-0007] Chotard, S. , Mason, J. B. , Oliphant, N. P. , Mebrahtu, S. , & Hailey, P. (2010). Fluctuations in wasting in vulnerable child populations in the Greater Horn of Africa. Food and Nutrition Bulletin, 31, S219–S233. 10.1177/15648265100313S302 21049843

[mcn12817-bib-0008] Christian, P. , Lee, S. E. , Donahue Angel, M. , Adair, L. S. , Arifeen, S. E. , Ashorn, P. , … Black, R. E. (2013). Risk of childhood undernutrition related to small‐for‐gestational age and preterm birth in low‐ and middle‐income countries. International Journal of Epidemiology, 42, 1340–1355. 10.1093/ije/dyt109 23920141 PMC3816349

[mcn12817-bib-0009] Costello, A. M. L. (1989). Growth velocity and stunting in rural Nepal. Arch Dis Childhood, 64, 1478–1482. 10.1136/adc.64.10.1478 2817933 PMC1792803

[mcn12817-bib-0033] de Onis, M. , & Branca, F. (2016). Childhood stunting: A global perspective. Maternal & Child Nutrition, 12(Suppl 1), 12–26. 10.1111/mcn.12231 27187907 PMC5084763

[mcn12817-bib-0010] Dewey, K. G. (2016). Reducing stunting by improving maternal, infant and young child nutrition in regions such as South Asia: Evidence, challenges and opportunities. Maternal & Child Nutrition, 12(Suppl 1), 27–38. 10.1111/mcn.12282 27187908 PMC5084734

[mcn12817-bib-0011] Dewey, K. G. , & Begum, K. (2011). Long‐term consequences of stunting in early life. Maternal & Child Nutrition, 7(Suppl 3), 5–18. 10.1111/j.1740-8709.2011.00349.x 21929633 PMC6860846

[mcn12817-bib-0012] Doherty, C. P. , Sarkar, M. A. K. , Shakur, M. S. , Ling, S. C. , Elton, R. A. , & Cutting, W. A. (2001). Linear and knemometric growth in the early phase of rehabilitation from severe malnutrition. Br Journ Nut, 85, 755–759. 10.1079/BJN2001351 11430781

[mcn12817-bib-0013] Galasso, E. , & Wagstaff, A. (2016). The economic costs of stunting and how to reduce them. Washington, D.C.: World Bank.

[mcn12817-bib-0014] Gat‐Yablonski, G. , Ben‐Ari, T. , Shtaif, B. , Potievsky, O. , Moran, O. , Eshet, R. , … Phillip, M. (2004). Leptin reverses the inhibitory effect of caloric restriction on longitudinal growth. Endocrinology, 145, 343–350. 10.1210/en.2003-0910 14525912

[mcn12817-bib-0015] Golden, M. H. (2009). Proposed recommended nutrient densities for moderately malnourished children. Food and Nutrition Bulletin, 30, S267–S342. 10.1177/15648265090303S302 19998863

[mcn12817-bib-0016] Goudet, S. , Griffiths, P. , Bogin, B. , & Madise, N. (2017). Interventions to tackle malnutrition and its risk factors in children living in slums: A scoping review. Annals of Human Biology, 44, 1–10. 10.1080/03014460.2016.1205660 27356853

[mcn12817-bib-0017] Grantham‐McGregor, S. , Cheung, Y. B. , Cueto, S. , Glewwe, P. , Richter, L. , & Strupp, B. (2007). Developmental potential in the first 5 years for children in developing countries. The Lancet, 369, 60–70. 10.1016/S0140-6736(07)60032-4 PMC2270351 17208643

[mcn12817-bib-0018] Grellety, E. , Shepherd, S. , Roederer, T. , Manzo, M. L. , Doyon, S. , Ategbo, E. A. , & Grais, R. F. (2012). Effect of mass supplementation with ready‐to‐use supplementary food during an anticipated nutritional emergency. PLoS ONE, 7, e44549 10.1371/journal.pone.0044549 22984524 PMC3440398

[mcn12817-bib-0019] Huybregts, L. , Houngbe, F. , Salpeteur, C. , Brown, R. , Roberfroid, D. , Ait‐Aissa, M. , & Kolsteren, P. (2012). The effect of adding ready‐to‐use supplementary food to a general food distribution on child nutritional status and morbidity: A cluster‐randomized controlled trial. PLoS Medicine, 9, e1001313 10.1371/journal.pmed.1001313 23028263 PMC3445445

[mcn12817-bib-0020] Isanaka, S. , Langendorf, C. , Berthe, F. , Gnegne, S. , Li, N. , Ousmane, N. , … Grais, R. F. (2016). Routine amoxicillin for uncomplicated severe acute malnutrition in children. The New England Journal of Medicine, 374, 444–453. 10.1056/NEJMoa1507024 26840134

[mcn12817-bib-0021] Isanaka, S. , Nombela, N. , Djibo, A. , Poupard, M. , Van Beckhoven, D. , Gaboulaud, V. , … Grais, R. F. (2009). Effect of preventive supplementation with ready‐to‐use therapeutic food on the nutritional status, mortality, and morbidity of children aged 6 to 60 months in Niger. Jama, 301, 277–285. 10.1001/jama.2008.1018 19155454 PMC3144630

[mcn12817-bib-0022] Isanaka, S. , Roederer, T. , Djibo, A. , Luquero, F. J. , Nombela, N. , Guerin, P. J. , & Grais, R. F. (2010). Reducing wasting in young children with preventive supplementation: a cohort study in Niger. Pediatrics, 126, e442–e450. 10.1542/peds.2009-2814 20660552 PMC3144628

[mcn12817-bib-0023] Karsenty, G. (2006). Convergence between bone and energy homeostases: Leptin regulation of bone mass. Cell Metabolism, 4, 341–348. 10.1016/j.cmet.2006.10.008 17084709

[mcn12817-bib-0024] Kerac, M. , Bunn, J. , Chagaluka, G. , Bahwere, P. , Tomkins, A. , Collins, S. , & Seal, A. (2014). Follow‐up of post‐discharge growth and mortality after treatment for severe acute malnutrition (FuSAM study): A prospective cohort study. PLoS ONE, 9, e96030 10.1371/journal.pone.0096030 24892281 PMC4043484

[mcn12817-bib-0025] Khara, T. , Dolan, C. (2014). The relationship between wasting and stunting, policy, programming and research implications. Emergency Nutrition Network USAID.

[mcn12817-bib-0026] Khara, T. , Mwangome, M. , Ngari, M. , & Dolan, C. (2017). Children concurrently wasted and stunted: A meta‐analysis of prevalence data of children 6‐59 months from 84 countries. Maternal & Child Nutrition, 14(2), e12516. 10.1111/mcn.12516 PMC5901398 28944990

[mcn12817-bib-0027] Lelijveld, N. , Seal, A. , Wells, J. C. , Kirkby, J. , Opondo, C. , Chimwezi, E. , … Kerac, M. (2016). Chronic disease outcomes after severe acute malnutrition in Malawian children (ChroSAM): a cohort study. The Lancet Global Health, 4, e654–e662. 10.1016/S2214-109X(16)30133-4 27470174 PMC4985564

[mcn12817-bib-0028] Luby, S. P. , Rahman, M. , Arnold, B. F. , Unicomb, L. , Ashraf, S. , Winch, P. J. , … Colford, J. M. Jr. (2018). Effects of water quality, sanitation, handwashing, and nutritional interventions on diarrhoea and child growth in rural Bangladesh: A cluster randomised controlled trial. The Lancet Global Health, 6, e302–e315. 10.1016/S2214-109X(17)30490-4 29396217 PMC5809718

[mcn12817-bib-0029] Maleta, K. M. , Virtanen, S. M. , Espo, M. , Kulmala, T. , & Ashorn, P. (2003). Seasonality of growth and the relationship between weight and height gain in children under three years of age in rural Malawi. Acta Paediatrica, 92, 491–497. 12801119 10.1111/j.1651-2227.2003.tb00584.x

[mcn12817-bib-0030] Martorell, R. , & Young, M. F. (2012). Patterns of stunting and wasting: Potential explanatory factors. Advances in Nutrition, 3, 227–233. 10.3945/an.111.001107 22516733 PMC3648726

[mcn12817-bib-0031] McDonald, C. M. , Olofin, I. , Flaxman, S. , Fawzi, W. W. , Spiegelman, D. , Caulfield, L. E. , … Nutrition Impact Model Study (2013). The effect of multiple anthropometric deficits on child mortality: Meta‐analysis of individual data in 10 prospective studies from developing countries. The American Journal of Clinical Nutrition, 97, 896–901. 10.3945/ajcn.112.047639 23426036

[mcn12817-bib-0032] Null, C. , Stewart, C. P. , Pickering, A. J. , Dentz, H. N. , Arnold, B. F. , Arnold, C. D. , … Colford, J. M. Jr. (2018). Effects of water quality, sanitation, handwashing, and nutritional interventions on diarrhoea and child growth in rural Kenya: A cluster‐randomised controlled trial. The Lancet Global Health, 6, e316–e329. 10.1016/S2214-109X(18)30005-6 29396219 PMC5809717

[mcn12817-bib-0034] Popkin, B. M. , Richards, M. K. , & Montiero, C. A. (1996). Stunting is associated with overweight in children of four nations that are undergoing the nutrition transition. The Journal of Nutrition, 126, 3009–3016. 10.1093/jn/126.12.3009 9001368

[mcn12817-bib-0035] Prendergast, A. J. , & Humphrey, J. H. (2014). The stunting syndrome in developing countries. Paediatr Int Child Health, 34, 250–265. 10.1179/2046905514Y.0000000158 25310000 PMC4232245

[mcn12817-bib-0036] Richard, S. A. , Black, R. E. , & Checkley, W. (2012). Revisiting the relationship of weight and height in early childhood. Advances in Nutrition, 3, 250–254. 10.3945/an.111.001099 22516736 PMC3648729

[mcn12817-bib-0037] Richard, S. A. , Black, R. E. , Gilman, R. H. , Guerrant, R. L. , Kang, G. , Lanata, C. F. , … Childhood Infection and Malnutrition Network (2012). Wasting is associated with stunting in early childhood. The Journal of Nutrition, 142, 1291–1296. 22623393 10.3945/jn.111.154922 PMC3374667

[mcn12817-bib-0038] Subramanian, S. V. , Mejia‐Guevara, I. , & Krishna, A. (2016). Rethinking policy perspectives on childhood stunting: Time to formulate a structural and multifactorial strategy. Maternal & Child Nutrition, 12(Suppl 1), 219–236. 10.1111/mcn.12254 27187918 PMC5084745

[mcn12817-bib-0039] Thakwalakwa, C. M. , Ashorn, P. , Jawati, M. , Phuka, J. C. , Cheung, Y. B. , & Maleta, K. M. (2012). An effectiveness trial showed lipid‐based nutrient supplementation but not corn‐soya blend offered a modest benefit in weight gain among 6‐ to 18‐month‐old underweight children in rural Malawi. Public Health Nutrition, 15, 1755–1762. 10.1017/S1368980012003023 22691922

[mcn12817-bib-0040] Walker, S. P. , & Golden, M. H. (1988). Growth in length of children recovering from severe malnutrition. Eur J Clin Nut, 42, 395–404. 3135181

[mcn12817-bib-0041] Walker, S. P. , Grantham‐McGregor, S. , Himes, J. H. , & Powell, C. A. (1996). Relationships between wasting and linear growth in stunted children. Acta Paediatrica, 85, 666–669. 10.1111/j.1651-2227.1996.tb14120.x 8816200

[mcn12817-bib-0042] Waterlow, J. C. (1974). Some aspects of childhood malnutrition as a public health problem. BMJ, 4, 88–90. 10.1136/bmj.4.5936.88 4213384 PMC1612178

[mcn12817-bib-0043] WHO (2014) WHA global nutrition targets 2025: Stunting policy brief. WHO.

[mcn12817-bib-0044] World Bank (2014) Reaching the global target to reduce stunting: How much will it cost and how can we pay for it?: World Bank.

