# Supplementary material for: Linear growth faltering and the role of weight attainment: Prospective analysis of young children recovering from severe wasting in Niger
Source: Matern Child Nutr. 2019 Apr 29;15(4):e12817. doi: 10.1111/mcn.12817 (PMC6849732; doi:10.1111/mcn.12817)
Supplement: Supplementary file 1 — Table S1. Mean change in weight‐for‐height Z and height‐for‐age Z during and after treatment among boys and girls 6–23 months and 24–59 months of age [file MCN-15-e12817-s001.docx]

|  | Change during treatment  Mean (95% CI) | Change after treatment  Mean (95% CI) |
| --- | --- | --- |
| **Weight-for-height Z** |  |  |
| Boys |  |  |
| 6-23 months | 0.34 (0.32, 0.35) | 0.02 (0.01, 0.03)* |
| 24-59 months | 0.38 (0.35, 0.42) | 0.03 (0.01, 0.05)* |
|  |  |  |
| Girls |  |  |
| 6-23 months | 0.29 (0.28, 0.31) | 0.03 (0.02, 0.04)* |
| 24-59 months | 0.35 (0.31, 0.40) | 0.04 (0.03, 0.06)* |
|  |  |  |
| **Height-for-age Z** |  |  |
| Boys |  |  |
| 6-23 months | -0.04 (-0.05, -0.04) | -0.01 (-0.02, -0.01)* |
| 24-59 months | -0.01 (-0.01, -0.01) | -0.00 (-0.01, 0.00) |
|  |  |  |
| Girls |  |  |
| 6-23 months | -0.05 (-0.06, -0.05) | -0.02 (-0.02, -0.01)* |
| 24-59 months | -0.01 (-0.02, -0.00) | 0.00 (-0.01, 0.01) |

Supplemental Table 1. Mean change in weight-for-height Z and height-for-age Z during and after treatment among boys and girls 6-23 months and 24-59 months of age

* P for difference between change in pre-treatment vs. change in post-treatment < 0.05
