# Supplementary figures and images for: Linear growth faltering and the role of weight attainment: Prospective analysis of young children recovering from severe wasting in Niger
Source: Matern Child Nutr. 2019 Apr 29;15(4):e12817. doi: 10.1111/mcn.12817 (PMC6849732; doi:10.1111/mcn.12817)

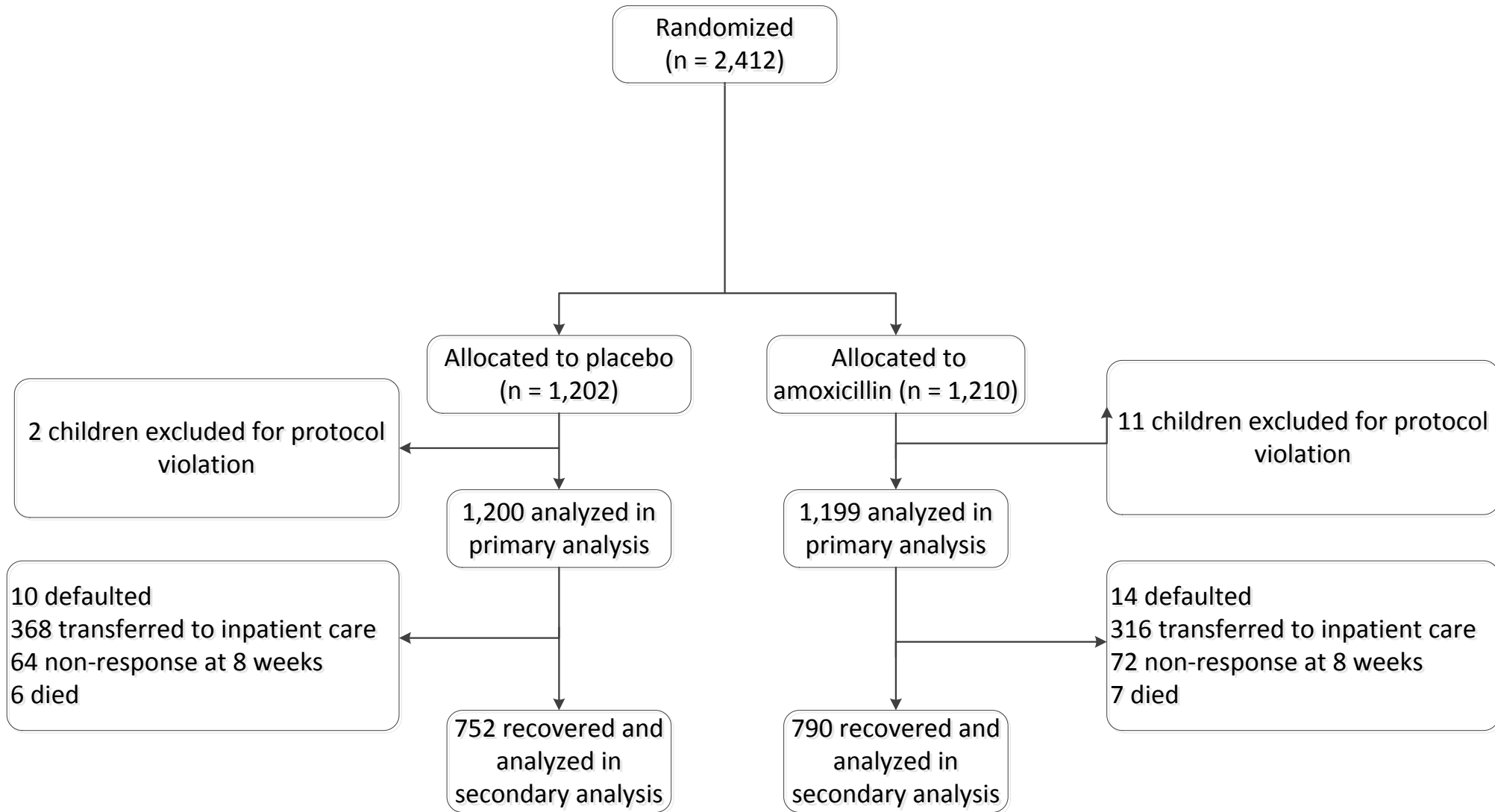

Supplement: Supplementary file 2 — Data S1. Supporting information [file MCN-15-e12817-s002.pdf]
